# Adolescent Responses to Coercive Requests for Sexual Images

**DOI:** 10.1001/jamanetworkopen.2026.0739

**Published:** 2026-03-17

**Authors:** Lisa M. Jones, Kimberly J. Mitchell, Deirdre Colburn, Ateret Gewirtz-Meydan

**Affiliations:** 1 Crimes Against Children Research Center, University of New Hampshire, Durham; 2 School of Social Work, Faculty of Social Welfare and Health Sciences, University of Haifa, Haifa, Israel

## Abstract

**Importance:**

Requests for sexual images are a common but understudied component of adolescent digital interactions. Although image sharing or sexting can be a consensual behavior between peers, when requests for sexual images are coercive it is a form of sexual harassment.

**Objective:**

To examine experiences with coercive requests for sexual images received before the age of 18 years and the incident and respondent characteristics associated with sharing an image in response to coercion.

**Design, Setting, and Participants:**

This survey study was conducted in the US between June 28, 2023, and April 1, 2024. Survey participants were a sample of 6204 young adults (aged 18-28 years). Screening procedures were designed to oversample individuals with histories of image-based sexual abuse to examine questions related to incident dynamics. Analyses used statistical weighting to improve generalizability to the US population.

**Main Outcomes and Measures:**

Bivariate and multivariate logistic regression modeling examined whether demographic and incident-level factors were associated with a decision by respondents to share a sexual image in response to coercion, defined as threats or strong pressure, and the impact of sharing on disclosure and mental health outcomes.

**Results:**

A total of 2853 respondents reported 4205 incidents of image-based sexual abuse, including 2003 coercive requests for sexual images that occurred before they were 18 years of age; in 1886 (mean respondent age, 22.8 [95% CI, 22.6-23.1] years; 91.0% female [95% CI, 89.4-92.4]) of these incidents, respondents provided information on whether they did or did not share an image in response, making up the analytic sample for the study. In 1067 coercive request incidents (55.6%; 95% CI, 51.5%-59.6%), adolescents shared an image in response. Sharing was significantly less likely when the perpetrator was someone the adolescent met online vs a dating partner (odds ratio [OR], 0.33; 95% CI, 0.20-0.54; *P* < .001) and was more likely for incidents lasting longer than month (OR, 3.55; 95% CI, 2.44-5.16; *P* < .001). Respondents who shared images reported significantly worse mental health outcomes than those who did not (OR, 1.10; 95% CI, 1.04 -1.17; *P* = .001) and were less likely to disclose the incident (OR, 0.69; 95% CI, 0.48-0.98; *P* = .04).

**Conclusions and Relevance:**

In this survey study of young adults in the US, findings identified significant harms associated with providing sexual images in response to coercive requests during adolescence. Results highlight the importance of providing adolescents with skills to respond to image requests and information about the harmful outcomes of coercive sexual image requests.

## Key Points

**Question:**

What incident and youth characteristics are associated with responses to coercive requests for images?

**Findings:**

In this survey study of 6204 young adults, the risk of sharing sexual images before the age of 18 years in response to coercive requests was significantly higher when requests were made by dating partners. Sharing an image in response to coercion was associated with reduced disclosure rates despite significantly greater negative outcomes for adolescents.

**Meaning:**

Prevention messages for adolescents should highlight the harm caused by coercive requests for sexual images and increase help-seeking options.

## Teaser

This survey study evaluates what incident and youth factors are associated with an adolescent's decision to share sexual images after coercive requests.

## Introduction

Sexual image sharing has become increasingly prevalent in youth digital culture.^1^ One meta-analysis found adolescent prevalence rates of 19.3% for sending sexual photos and 34.8% for receiving, with females more frequently receiving requests and older adolescents more likely to engage in sending behaviors. Many respondents perceive sexual image sharing as a normal part of dating^2,3^ and see sexting as normative among their peers.^4,5^ Although requesting and sharing sexual images can be consensual in the context of romantic relationships, there are risks for adolescents. Sexual images may be shared without consent and threats to share the images may be used coercively after a relationship ends.^6,7^ Such cases are increasingly recognized as forms of image-based sexual abuse (IBSA).^8,9^ One study^10^ found that more than one-third of adolescents who shared sexual images reported adverse outcomes. Coercive requests for images can also be distressing experiences for adolescents and increase the chance that the exchange may not be fully consensual.^11^ Even in the absence of overt threats, many adolescents experience strong pressure to send intimate images, pressure that can be emotional, manipulative, or persistent in nature.^12,13,14^ Coercive requests for sexual images can be considered an online form of sexual harassment, which research has linked to adverse outcomes, such as sleep problems, depressive symptoms, suicidal ideation, and substance use for adolescents.^15^

It is important to understand the circumstances under which adolescents feel compelled to share a sexual image in response to coercive requests to effectively target prevention and intervention programs. Prior research has found that when asked to send a sexual image by a current boyfriend or girlfriend, 70% of adolescents comply,^2^ but it is not clear how they respond when a request is coercive. Although some may refuse to share images, others may comply due to feeling scared, ashamed, or concerned about the relational consequence of refusing.^12,16^ The current study examines experiences with coercive requests for sexual images received before the age of 18 years reported by a large sample of young adult survey respondents and the incident and respondent characteristics associated with sharing an image in response to coercion. The study also assesses the differential association of sharing vs not sharing with disclosure behavior and outcome of the incident for adolescents.

## Methods

### Study Design

The survey study was conducted between June 28, 2023, and April 1, 2024, in the US. A sample of 6204 young adults (aged 18-28 years) was recruited through Facebook and Instagram advertisements promoting an opportunity to share the experiences of young people in survey research. Three screening questions (measured on a 5-point Likert scale) were administered before survey administration, asking whether the respondent had ever sent a sexual image or video, been asked for a sexual image or video, or talked with friends about the risks of sending sexual images or videos. The purpose of the screening questions was to ensure an oversample of individuals with IBSA experiences. Study involvement of human participants and informed consent procedures were approved by the University of New Hampshire Institutional Review Board. All participants provided written consent to participate. The study followed the American Association for Public Opinion Research (AAPOR) reporting guidelines.

Eligible respondents were routed to an online survey and those who completed the survey and provided a valid US non–PO box mailing address were sent a $15 Amazon gift card. Requiring a physical US mailing address for the gift card successfully deterred fraudulent entries from outside the US, a common challenge with online recruitment strategies that offer an email incentive.^17^ Additional steps were taken to deter and detect any fraudulent entries, including sending one incentive per address, using Qualtrics settings to limit multiple responses and detect duplicate and fraudulent entries (eg, RECAPTCHA Score, RelevantID Duplicate Score, and RelevantID Fraud Score), attention-check items in the survey, and reviewing responses for completion time, conflicting response patterns, and straight-lining.

### Measures

Survey items were adapted from a prior study^9^ on technology-facilitated abuse. Outcome measures were demographic and incident-level factors associated with a decision by respondents to share a sexual image in response to coercion, defined as threats or strong pressure, and the outcome of sharing for disclosure and mental health.

#### Respondent Characteristics

Survey respondents reported on their age at the time of the incident (≤12, 13-15, or 16-17 years), age at the time of the survey (continuous); gender, sexual orientation, race and ethnicity, and whether they had received a high school diploma. Self-report data on race and ethnicity were collected to characterize the sample and to evaluate whether outcome variables varied across groups, recognizing that structural inequities may shape adolescents’ vulnerability to online sexual harm and access to protective resources. Additionally, respondents were asked about 11 types of adversity (eg, living on the street or in a shelter, being sent or taken away from your family, or knew someone who overdosed on prescription medication pills or other drugs) and 8 types of abuse or maltreatment (eg, an adult hitting them) that they may have experienced before 18 years of age. Counts of all adversities and types of abuse or maltreatment were included in analyses.

#### Incident Characteristics

Respondents were asked how long the incident lasted (≥1 vs <1 month), how many times requests for images were made (≤3 vs ≥4), and the number of perpetrators (1 vs ≥2). Information about the perpetrator was coded as adult (vs minor or unknown) and relationship between the respondent and perpetrator (dating partner, friend or acquaintance, or stranger or person known only online). Respondents were also asked whether a threat was involved in the request for images (yes or no).

#### Incident Outcomes and Impact

Respondents were asked whether they had told anyone about the incident before they were 18 years of age (yes or no) and about the degree the event impacted them (dichotomized as impacting them little or somewhat vs significantly). Respondents were also asked their perspectives on whether the event negatively affected them in 16 different ways (eg, getting worse grades, trouble concentrating, or thinking about hurting themselves). Items were analyzed individually (yes or no), and a composite variable was created as a sum of the different ways the event impacted them (scale of 0-16, with 0 indicating negative outcomes and 16 indicating positive outcomes). Lastly, depressive symptoms in the past 30 days were measured using the Kessler Psychological Distress Scale (K10).^18^ Response options ranged from 1 (none of the time) to 5 (all of the time). Items were combined for a total scale score with higher values indicating more symptoms. The reliability of the scale in the current study was good (α = .82). For analyses, high depression was coded as scores greater than 1 SD.

### Statistical Analysis

Data were weighted to general population targets using statistics from the Current Population Survey^19^ for individuals aged 18 to 28 years on age, gender, educational level, race and Hispanic ethnicity, and gender identity and sexual identity (from Pew Research).^20,21^ Unweighted sample sizes are reported; weighted percentages and 95% CIs are provided. Missing data were less than 5% for all variables. For categorical variables, missing and prefer not to answer responses are provided in tables. For dummy-coded variables, missing data were coded as not present. For continuous variables mean replacement was used for missing data.

Analyses were conducted using Stata SE, version 19 (StataCorp).^22^ Unadjusted logistic regression analyses were conducted to examine bivariate differences between respondents who did and did not share sexual images in response to coercive requests across respondent characteristics, incident characteristics, and incident outcomes. Multivariate logistic regression models included all variables with bivariate associations statistically significant at a 2-sided *P* < .10 and estimated adjusted odds ratios (ORs) for respondent and incident characteristics on decision to share image in response to coercion. Two-sided *P* < .05 was considered statistically significant.

## Results

Of 6204 respondents completing the survey, 2853 respondents reported on a total of 4205 incidents of image-based sexual abuse, including 2003 coercive requests for sexual images (involving threats, force, or strong pressure) that occurred before they were 18 years of age. In 1886 of these incidents (mean respondent age, 22.8 [95% CI, 22.6-23.1] years; 91.0% female [95% CI, 89.4%-92.4%]; 13.6% Black [95% CI, 10.5%-17.3%], 64.1% White [95% CI, 60.0%-68.0%], 4.7% multiple [95% CI, 3.8%-5.9%], and 12.0% other [95% CI, 9.6%-15.0%], including American Indian or Alaska Native, Asian, Hawaiian or Pacific Islander, and other), respondents provided information on whether they did or did not share an image in response, making up the analytic sample for the study. In 1067 coercive request incidents (55.6%; 95% CI, 51.5%-59.6%), respondents shared an image in response. Sexual orientation before 18 years of age was reported as heterosexual by 68.6% of the sample (95% CI, 65.3%-71.8%). Most respondents reported receiving a high school diploma at the time of the survey (78.5%; 95% CI, 74.7%-81.9%), and 43.2% (95% CI, 33.5%-41.3%) reported having a family with a lower-than-average household income. Table 1 provides weighted demographic distributions for the full sample.

**Table 1.** Bivariate Associations Between Respondent Characteristics and Image Sharing in Response to Coercion

Respondent characteristic	Incidents, weighted % (95% CI)^a^			OR (95% CI)	*P* value
	All incidents (N = 1886)	Did not share image (n = 819)	Shared image (n = 1067)		
Age at incident, y					
≤12	11.7 (9.5-14.3)	13.6 (9.99-18.1)	10.2 (7.6-13.5)	1.00 [Reference]	NA
13-15	48.5 (44.4-52.6)	46.4 (40.4-52.5)	50.2 (44.7-55.6)	1.44 (0.88-2.34)	.15
16-17	38.8 (33.5-44.3)	38.9 (32.9-45.2)	38.8 (33.5-44.3)	1.32 (0.79-2.22)	.29
Missing or PNA	1.0 (0.5-1.8)	1.2 (0.4-3.1)	0.8 (0.4-1.7)	0.95 (0.26-3.48)	.93
Gender (aged <18 y)					
Female	91.0 (89.4-92.4)	91.0 (88.4-93.1)	91.0 (88.9-92.7)	1.00 [Reference]	NA
Male	1.0 (0.6-1.4)	0.6 (0.3-1.0)	1.2 (0.7-2.1)	2.11 (0.97-4.62)	.06
Other^b^	7.3 (6.0-8.8)	7.5 (5.6-10.1)	7.1 (5.5-9.0)	0.94 (0.62-1.42)	.78
Missing or PNA	0.8 (0.6-1.1)	0.9 (0.5-1.4)	0.7 (0.5-1.1)	0.84 (0.43-1.63)	.44
Sexual orientation (aged <18 y)					
Heterosexual	68.6 (65.3-71.8)	73.3 (68.7-77.4)	64.9 (60.1-69.5)	1.00 [Reference]	NA
Bisexual	19.9 (17.3-22.8)	13.7 (11.0-17.0)	24.8 (20.8-29.3)	2.04 (1.43-2.92)	<.001
Other^c^	9.3 (8.0-10.84)	9.8 (7.8-12.2)	9.0 (7.3-11.0)	1.03 (0.72-1.47)	.86
Missing or PNA	2.1 (1.3-3.5)	3.2 (1.6-6.1)	1.3 (0.7-2.5)	0.46 (0.17-1.20)	.33
Race					
Black	13.6 (10.5-17.3)	14.4 (9.8-20.5)	12.9 (9.1-18.0)	1.00 [Reference]	NA
White	64.1 (60.0-68.0)	61.5 (55.1-67.4)	66.2 (60.7-71.2)	1.20 (0.66-2.16)	.55
Other^d^	12.0 (9.6-15.0)	12.7 (9.1-17.5)	11.4 (8.4-15.4)	1.00 (0.48-2.08)	>.99
Multiple	4.7 (3.8-5.9)	4.8 (3.4-6.8)	4.6 (3.5-6.1)	1.07 (0.53-2.17)	.85
Missing or PNA	5.6 (4.0-7.8)	6.6 (4.2-10.4)	4.8 (3.0-7.6)	0.81 (0.34-1.93)	.63
High school diploma	78.5 (74.7-81.9)	82.4 (76.9-86.9)	75.4 (70.1-80.0)	0.06 (0.42-1.01)	.055
Income lower than average	37.4 (33.5-41.3)	39.1 (33.3-45.2)	36.0 (31.0-41.3)	0.88 (0.63-1.23)	.44
Rural community	29.2 (25.6-33.1)	29.3 (23.9-35.3)	29.2 (24.4-34.5)	0.99 (0.69-1.44)	.98
Age at survey, mean (95% CI), y	22.8 (22.6-23.1)	22.7 (22.3-23.1)	22.9 (22.6-23.2)	1.02 (0.97-1.08)	.42
Total abuse score (aged <18 y), mean (95% CI)	3.66 (3.49-3.83)	3.66 (3.40-3.92)	3.66 (3.43-3.89)	1.00 (0.92-1.08)	.92
Total adversity score (aged <18 y), mean (95% CI)	3.49 (3.29-3.69)	3.31 (3.02-3.60)	3.64 (3.36-3.91)	1.06 (0.99-1.07)	.10

Abbreviations: NA, not applicable; OR, odds ratio; PNA, prefer not to answer.

^a^ Data are weighted to approximate US population.

^b^ Other gender category included transgender, genderqueer, and other identity.

^c^ Other sexual orientation category included gay, lesbian, and other identity.

^d^ Other racial category included Asian, Hawaiian or Pacific Islander, American Indian or Alaska Native, and other identity.

### Youth and Incident Characteristics and Response to Coercive Image Request

Respondent demographics, such as age at the time of the incident, gender identity, race and ethnicity, educational level, income level, rural vs urban residence, and age at time of survey, were not significantly associated with response to coercive requests for sexual images (Table 1). Sexual orientation was a significant factor: adolescents who shared an image were more likely to report a bisexual sexual orientation compared with those who did not share an image (OR, 2.04; 95% CI, 1.43-2.92; *P* < .001). There were no significant differences in response based on the total number of adversities or experiences of violence reported before 18 years of age.

Several incident characteristics were significantly associated with the likelihood of image sharing in response to coercion (Table 2). The number of perpetrators and the age of the perpetrator (youth vs adult) were not associated with whether an image was shared by the respondents in response to coercion. Respondents were significantly less likely to have shared an image when the perpetrator was an acquaintance (OR, 0.22; 95% CI, 0.13-0.39; *P* < .001) or someone known only online (OR, 0.33; 95% CI, 0.20-0.54; *P* < .001) compared with a dating partner. Sharing an image was more likely if the coercive requests lasted more than 1 month (OR, 3.55; 95% CI, 2.44-5.16) and when requests had been made 4 or more times (OR, 2.24; 95% CI, 1.59-3.14; *P* < .001). There was no statistically greater likelihood of sharing images in response to coercion when threats vs strong pressure was used.

**Table 2.** Bivariate Analysis of Incident Characteristics by Image Sharing in Response to Coercion

Incident characteristic	Incidents, weighted % (95% CI)^a^			OR (95% CI)	*P* value
	All incidents (N = 1886)	Did not share image (n = 819)	Shared image (n = 1067)		
≥2 Perpetrators	16.7 (13.9-20.0)	17.2 (13.0-22.3)	16.4 (12.8-20.7)	0.94 (0.61-1.45)	.78
Adult perpetrators	53.0 (48.9-57.0)	51.8 (45.7-57.9)	54.0 (48.6-59.3)	1.09 (0.78-1.51)	.60
Perpetrator relationship to respondent					
Dating partner	22.1 (18.9-25.7)	11.5 (8.0-16.3)	30.5 (25.8-35.7)	1.00 [Reference]	NA
Friend or acquaintance	21.7 (18.6-25.3)	28.0 (22.9-33.8)	16.7 (13.0-21.3)	0.22 (0.13-0.39)	<.001
Online only or stranger	53.4 (49.4-57.5)	57.3 (51.1-63.2)	50.4 (45.0-55.8)	0.33 (0.20-0.54)	<.001
Missing or PNA	2.7 (1.7-4.2)	3.2 (1.8-5.5)	2.3 (1.2-4.5)	0.28 (0.10-0.74)	.01
Happened ≥4 times	40.0 (36.0-43.9)	29.4 (24.3-35.1)	48.2 (42.8-53.7)	2.24 (1.59-3.14)	<.001
Incident lasted ≥1 month	31.1 (27.5-34.9)	17.1 (13.2-21.8)	42.2 (37.0-47.7)	3.55 (2.44-5.16)	<.001
Threat involved	13.1 (10.6-16.1)	11.3 (8.0-15.8)	14.5 (11.0-18.7)	1.32 (0.81-2.16)	.26

Abbreviation: NA, not applicable; OR, odds ratio; PNA, prefer not to answer.

^a^ Data are weighted to approximate US population.

### Image Sharing and Psychological Outcomes

Respondents who shared images in response to coercive image requests were significantly less likely to disclose the incident to someone before 18 years of age compared with those who did not provide an image (OR, 0.69; 95% CI, 0.48-0.98; *P* = .041) (Table 3). Respondents who shared images in response to coercive requests were also more likely to report that the outcome of the event for them was “significant” (OR, 2.89; 95% CI, 1.95-4.28; *P* < .001) and identify a greater number of negative psychological outcomes (OR, 1.10; 95% CI, 1.04-1.17; *P* = .001). The Figure displays the range of negative outcomes reported by respondents and the differences for respondents who provided an image. Respondents who provided an image were significantly more likely to report that the incident resulted in breaking up relationships (OR, 1.88; 95% CI, 1.15-3.07; *P* = .01), skipping school (OR, 1.67; 95% CI, 1.07-2.60; *P* = .02), thoughts about hurting self (OR, 2.72; 95% CI, 1.92-3.84; *P* < .001), seeing a physician or counselor (OR, 1.69; 95% CI, 1.07-2.67; *P* = .02), moving to a new house (OR, 3.55; 95% CI, 1.24-10.21; *P* = .02), and trouble with police (OR, 5.65; 95% CI, 1.82-17.55; *P* = .003) than adolescent who did not provide an image in response to coercion. There were no significant differences in depressive symptoms between the 2 groups at the time of survey administration.

**Table 3.** Bivariate Analysis of Incident Outcomes and Sexual Image Sharing in Response to Coercion

Outcome	Incidents, weighted % (95% CI)^a^			OR (95% CI)	*P* value
	All incidents (N = 1886)	Did not share image (n = 819)	Shared image (n = 1067)		
Disclosed incident (aged <18 y)	27.7 (24.3-31.4)	31.9 (26.6-37.6)	24.4 (20.1-29.3)	0.69 (0.48-0.98)	.04
Outcome of event for respondent reported as “significant”	25.8 (22.4-29.5)	15.3 (11.6-19.8)	34.2 (29.2-39.7)	(2.89 (1.95-4.28)	<.001
Sum of negative outcomes, mean (95% CI)	2.83 (2.60-3.07)	2.40 (2.09-2.69)	3.18 (2.84-3.53)	1.10 (1.04-1.17)	.001
High depressive symptoms (+1 SD)	14.2 (11.6-17.1)	14.6 (11.0-19.2)	13.8 (10.4-17.9)	0.93 (0.59-1.46)	.76

Abbreviation: OR, odds ratio.

^a^ Data are weighted to approximate US population.

**Figure.**
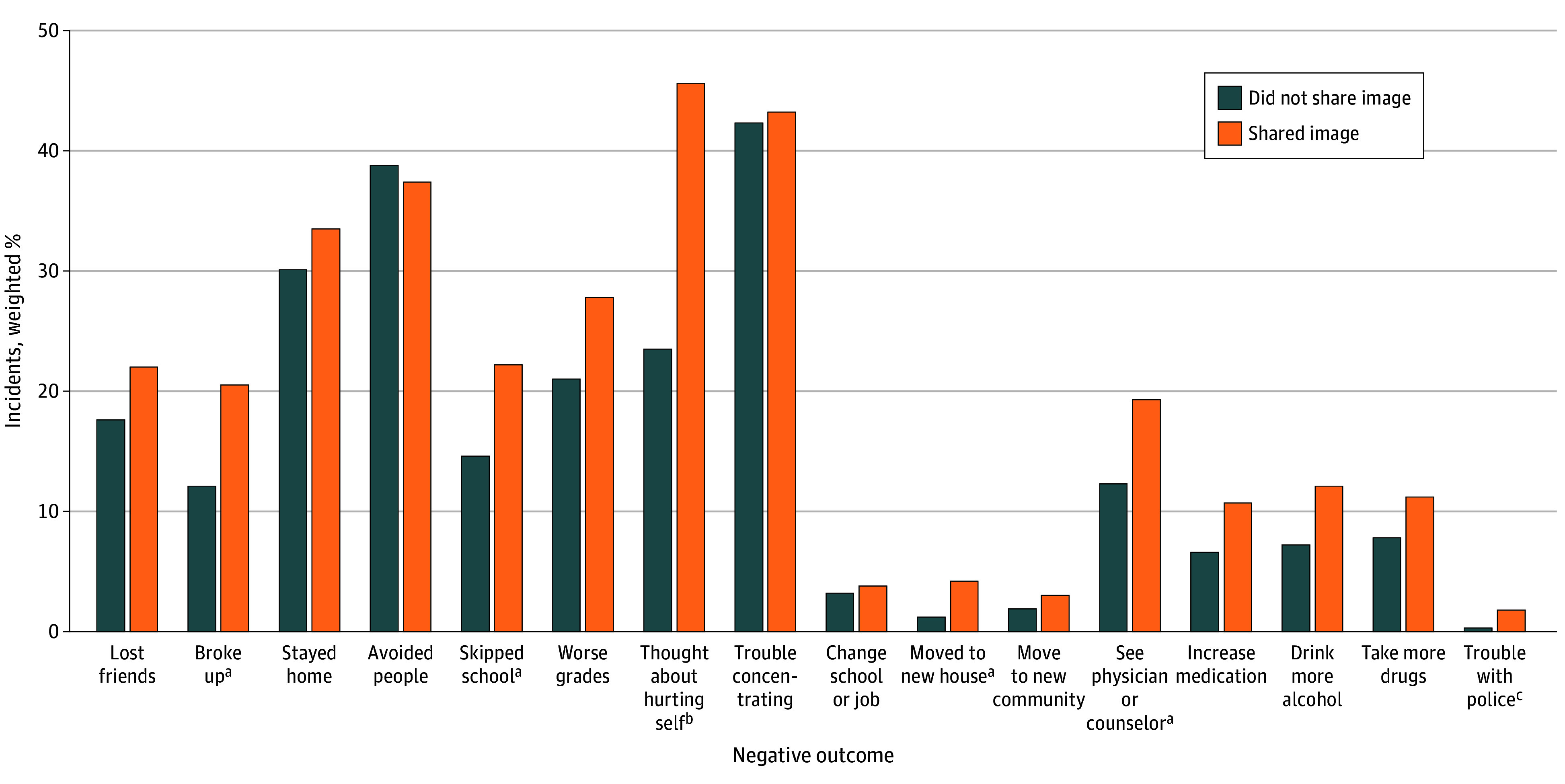
Negative Outcomes of Coercive Requests for Sexual Images by Decision to Share ^a^*P* < .05. ^b^*P* < .001. ^c^*P* < .01.

### Logistic Regression of Factors Associated With Response to Coercive Image Requests

A multivariate logistic regression model (Table 4) examined the unique contribution of factors associated with sharing an image in response to coercive requests that were significant at *P* < .10 at the bivariate level. Bisexual respondents were more likely to share a sexual image when coerced than heterosexual respondents (OR, 1.27; 95% CI, 1.05-2.24; *P* = .04). Respondents were significantly less likely to share an image when the perpetrator was a friend or acquaintance (OR, 0.23; 95% CI, 0.31-0.42; *P* < .001) or a stranger or someone known only online (OR, 0.35; 95% CI, 0.22-0.58; *P* < .001) compared with dating partners. Respondents were more likely to share a sexual image when the incident lasted 1 month or longer (OR, 2.48; 95% CI, 1.66-3.70; *P* = .001) and when image requests were made 4 or more times (OR, 1.89; 95% CI, 1.31-2.74; *P* < .001).

**Table 4.** Multivariate Logistic Regression on Decision to Share Images in Response to Coercive Requests

Factor	OR (95% CI)	*P* value
Gender		
Female	1.00 [Reference]	NA
Male	1.97 (0.83-4.71)	.13
Other	0.82 (0.48-1.39)	.46
Missing or PNA	0.70 (0.37-1.30)	.26
Respondent sexual orientation		
Heterosexual	1.00 [Reference]	NA
Bisexual	1.53 (1.05-2.24)	.03
Other	0.99 (0.64-1.53)	.98
Missing or PNA	0.48 (0.21-1.09)	.08
High school diploma	0.67 (0.42-1.08)	.10
Perpetrator relationship to respondent		
Dating partner	1.00 [Reference]	NA
Friend or acquaintance	0.23 (0.31-0.42)	<.001
Online only or stranger	0.35 (0.22-0.58)	<.001
Lasted ≥1 mo	2.48 (1.66-3.70)	.001
Requested ≥4 times	1.89 (1.31-2.74)	<.001

Abbreviations: NA, not applicable; OR, odds ratio; PNA, prefer not to answer.

## Discussion

Survey data from a large sample of young adult respondents found that after receiving a coercive request for a sexual image before the age of 18 years, more than half had complied by sharing an image. Respondents who sent sexual images in response to a coercive request were more likely to do so when the person making the request was a dating partner and when the requests involved threats, lasted 1 month or more, or were repetitive (≥4 times). Sharing an image was significantly associated with negative outcomes for respondents and with less disclosure and help-seeking compared with those who did not share images.

### Factors Associated With Sharing Sexual Images After Coercive Requests

Demographic differences and adversity histories largely did not distinguish respondents who did and did not share images in response to coercive requests. Respondents reporting their sexual orientation as bisexual were the only subgroup significantly more likely to have shared images in response to coercion, even after controlling for incident characteristics. There is currently limited research on IBSA experienced by sexual and gender minority adolescents, but some studies suggest heightened risk for these populations.^23,24^ Experiences of stigma and problematic connections with parents and peers may lead these adolescents to feel more isolated and less empowered when faced with coercion and sexual harassment.^25,26^

Public concern and prevention messaging around IBSA often focus on online strangers communicating with adolescents in deceptive ways.^27,28^ More than half of the coercive requests for images came from strangers or individuals known only online, with the rest coming from dating partners and acquaintances. However, respondents were significantly more likely to share sexual images in response to coercive requests from dating partners compared with strangers or acquaintances. Other research has similarly found that IBSA and sextortion are commonly perpetrated by partners and peers.^29,30^

### Outcomes of Sharing Sexual Images After Coercive Requests

The decision to share sexual photos in response to coercive requests was associated with significantly greater self-reported negative outcomes for respondents, negatively affecting their relationships and school attendance, increased suicidal ideation, and life disruptions. Prior research has similarly documented that coercive sexting negatively affects mental health and is associated with offline sexual coercion.^30,31,32,33^ Those experiencing IBSA report feeling ongoing fear over the circulation or resurfacing of their images online as well as worry about being recognized in public.^6,34,35^ Public accessibility of the images may also contribute to the feelings of ongoing vulnerability, helplessness, and powerlessness.

Study findings also identified a negative association between sharing a sexual image in response to coercion and disclosure. Adolescents who received coercive requests for sexual images but did not provide an image were more likely to disclose the experience to someone compared with those who provided an image. It may be that disclosing the coercive requests provided respondents with support and made it easier for them to avoid sharing an image. It also may be that respondents who share sexual images in response to coercion feel shame in ways that inhibit disclosure despite experiencing significant negative emotional outcomes. Prior research has found that shame generally inhibits disclosure for sexual abuse survivors^36^ and that shame and fear of negative reactions by others are central to the decision by survivors of IBSA to disclose or not disclose.^37,38^

### Implications for Prevention and Intervention

Prevention efforts are increasing to help adolescents identify online risk, including image-based abuses.^39^ There is evidence that sexual and relationship health programs can reduce sexual abuse.^40^ Findings from the current study suggest that dating partners hold more risk for adolescents, yet much of the current efforts focus on the risk of exchanging information with unknown individuals online.^40,41^ Generally, adolescents need to understand that pressure to share sexual images is harmful and that for those who feel compelled to share sexual images in response to coercion, the impacts on them can be substantial, affecting school work and mental health. Many adolescents who make these kinds of pressured, repeated requests, particularly in the context of dating relationships, may not understand that the impact of this behavior can be significantly negative and a form of sexual harassment and abuse. Adolescents should be provided with skills to help in refusal and resistance when they are feeling reluctant to share an image.^42,43^

### Limitations

Limitations of the study should be considered when interpreting findings. The study used online recruitment through social media, screening for adolescents who were more likely to have had experiences with IBSA. This recruitment strategy resulted in large percentages of LGBTQ+ adolescents and adolescents with IBSA histories. The study was not intended to provide nationally representative rates of IBSA but to understand the dynamics and outcomes of IBSA among young people who had experienced this form of abuse. However, findings may not represent the experiences of all adolescents in the US. Additionally, young adult survey respondents were reporting retrospectively on experiences they had when they were younger. For most of the respondents, this was only a few years since the incident described in the study; however, recall bias may have affected findings. Lastly, the study’s cross-sectional design prevents us from making conclusions about the long-term effects of sharing vs not sharing sexual images in response to coercion.

## Conclusions

In this survey study, 6204 young adults from across the US reported on 1886 incidents of coercive requests for sexual images that they received before 18 years of age. In more than half of the incidents, adolescents responded to the coercive requests by sharing images. Adolescents were more likely to provide images when the coercive request came from a dating partner or acquaintance, and when the requests occurred multiple times or over more than a month. Adolescents who shared images in response to coercive requests were much more likely than those who did not experience significant negative outcomes after the incident, including suicidal ideation, and were less likely to disclose the abuse to anyone. Findings highlight the importance of including information about pressured and coercive requests for images in prevention education programs.

## Notes

Supplement.
Data Sharing Statement
