# Supplementary material for: Adolescent Responses to Coercive Requests for Sexual Images
Source: JAMA Netw Open. 2026 Mar 17;9(3):e260739. doi: 10.1001/jamanetworkopen.2026.0739 (PMC13446802; doi:10.1001/jamanetworkopen.2026.0739)
Supplement: Supplementary file 1 [file jamanetwopen-e260739-s001.pdf]

Data Sharing Statement

Jones. Adolescent Responses to Coercive Requests for Sexual Images. *JAMA Netw Open*. Published March 17, 2026. doi:10.1001/jamanetworkopen.2026.0739

Data
Data available: Yes
Data types: Deidentified participant data
How to access data: Please contact lisa.jones@unh.edu to request data access.
When available: With publication

Supporting Documents
Document types: None

Additional Information
Who can access the data: researchers whose proposed use of the data has been approved
Types of analyses: research
Mechanisms of data availability: without investigator support
Any additional restrictions: none
